# Nanofibrous Photothermal Materials from Natural Resources: A Green Approach for Artwork Restoration

**DOI:** 10.1021/acsami.4c14532

**Published:** 2024-12-06

**Authors:** Arianna Menichetti, Francesca Ramacciotti, Giorgia Sciutto, Maria Letizia Focarete, Marco Montalti, Silvia Prati, Chiara Gualandi

**Affiliations:** 1 Department of Chemistry “Giacomo Ciamician”, University of Bologna, Via Selmi 2, Bologna 40126, Italy; 2 INSTM UdR of Bologna, University of Bologna, Via Selmi 2, Bologna 40126, Italy; 3 Department of Chemistry “Giacomo Ciamician”, University of Bologna, Via Dario Campana 71, Rimini 47922, Italy; 4 Health Sciences & Technologies (HST) CIRI, University of Bologna, Via Tolara di Sopra 41/E, Ozzano Emilia Bologna 40064, Italy; 5 Interdepartmental Center for Industrial Research on Advanced Applications in Mechanical Engineering and Materials Technology, CIRI-MAM, University of Bologna, Viale Risorgimento, 2, Bologna 40136, Italy

**Keywords:** electrospinning, polysaccharide, melanin, photothermal action, art conservation

## Abstract

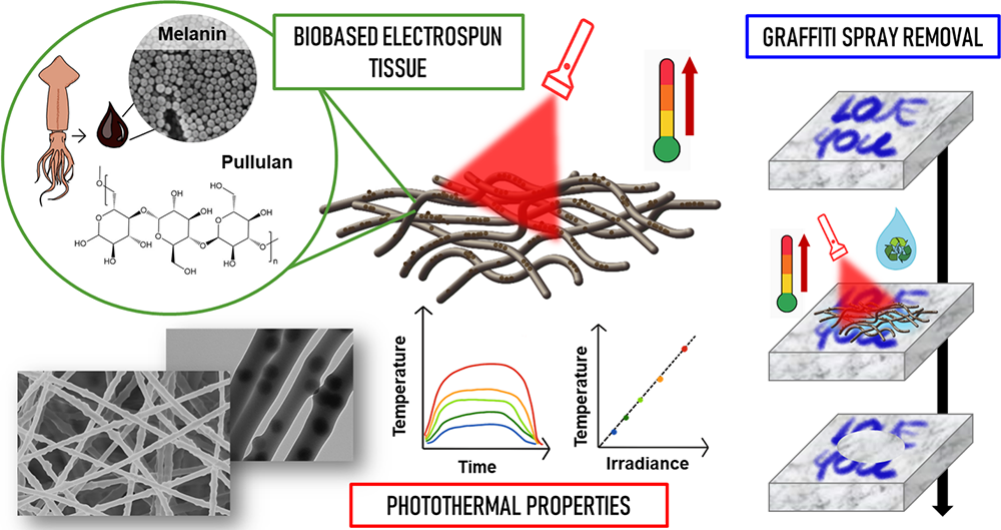

Cleaning unwanted paint layers represents a significant challenge in cultural heritage restoration, requiring high effectiveness, spatial precision, and nontoxic techniques. Cleaning vandalic acts or street art paints is particularly challenging because of insoluble varnishes, which are very resistant to traditional removal treatments. Here, for the first time, we employ the photothermal effect for cleaning an artwork, using electrospun nonwovens incorporated with melanin nanoparticles (NPs). This material shows outstanding photothermal properties and photostability. The nonwoven incorporated with melanin NPs, in combination with a solvent, efficiently removes alkyd resin paint layers in a short time of application, with high spatial control. Moreover, an eco-compatible system is obtained by producing a nonwoven made up of a natural polymer electrospun in water, cuttlefish ink as a melanin source, and a green solvent. In summary, using the new pullulan–melanin nonwoven represents a novel and unusual application of the photothermal effect, and its fastness, effectiveness, and safety make it suitable for use in the artwork restoration field.

## Introduction

In recent years, researchers have developed a growing interest in designing light-responsive materials in which a light stimulus controls a chemical or physical modification.^1,2^ In this context, photothermal agents are a class of molecules and materials in which light irradiation induces a temperature increase. After light excitation, these species undergo a nonradiative deactivation pathway that releases heat. Currently, the photothermal effect is widely employed in nanomedicine for photothermal therapy^3^ and antibacterial wound dressing,^4^ but it is also used in energy conversion and environmental technologies.^5^

An innovative application of the photothermal effect may be in art restoration. Here, the hypothesis is to exploit the light-induced localized increase in temperature to accelerate and improve the solubilization/swelling of hardly removable coatings and varnishes used in street art or vandalic acts. In particular, spray varnishes employed in graffiti are often made of alkyd resins whose removal represents a challenge since these varnishes consist of mixtures of monomers of polyesters, polyols, fatty acids and drying oils that, after application, polymerize to form a highly insoluble cross-linked polymer film.^6^

Nowadays, these paints are removed using chemical methods, such as the application of organic solvents (acetone, alcohols) or alkali caustic solvents,^7^ and physical methods based on scalpel, abrasive dust, pressurized water and sandblasting.^8^ Physical methods are known to yield inhomogeneous results and may damage the substrate surface.^9^ On the other hand, chemical methods involve a consistent and prolonged use of solvents applied by soft brush or cotton swabs that may damage the treated surface and pose risks for the environment and the user’s safety.^8^ Biological treatments, which exploit microorganisms’ action,^10^ have also been proposed but are still under development.^7^ Laser ablation has also been applied to remove graffiti, especially in historic buildings and structures.^11,12^ However, laser cleaning is not easily applicable on a broad range of substrates and types of varnishes. Indeed, it was observed that on some types of stones it can damage the microcrystalline structure and cause yellowing,^13^ while it can occur that the parameters need to be adjusted case by case depending also on the color of the spray paint.^14,15^ It is worth mentioning that laser-cleaning is more expensive than traditional methods.^8^

In art restoration, innovative cleaning methods used for the selective removal of unwanted layers, such as coatings or vandalic acts, employ solvent-retaining agents to control the release of solvents on the surface being treated.^16−19^ Besides using gels,^20,21^ we have recently proposed the use of electrospun nanofibers to remove terpenic varnishes from paintings.^22,23^ Electrospinning is a method to fabricate exceedingly thin fibers by applying electrostatic forces to elongate a viscoelastic jet originating from a polymer solution.^24,25^ Polymer nanofibers are characterized by high porosity, large specific surface area, and high aspect ratio and are employed in many fields,^26,27^ such as drug delivery,^28^ wound healing,^29^ tissue engineering,^30^ and reinforcement in composite materials.^31^ In painting cleaning, the electrospun nonwovens are soaked with a solvent and put in contact with the area to be cleaned. Besides working as solvent-retaining scaffolds, they also act as adsorbent agents. In this way, the swelled varnish can be peeled off from the substrate without additional mechanical action.^23^

**Figure 1:**
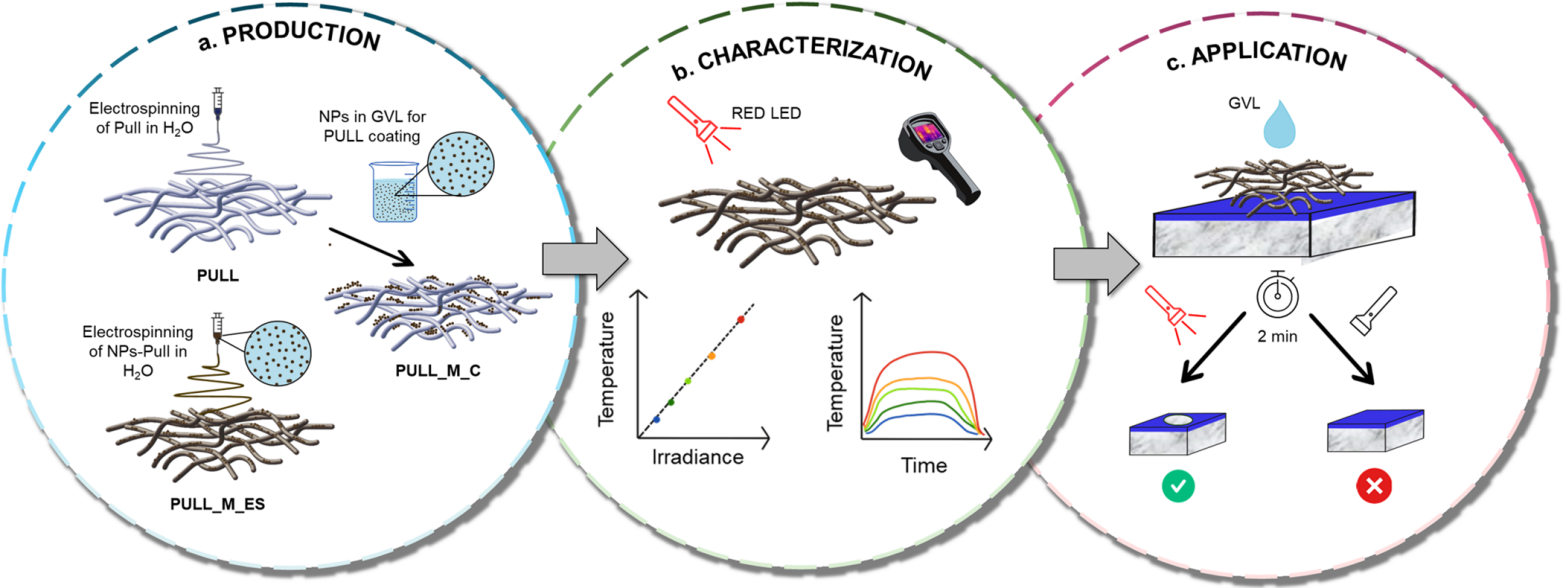
Scheme of production (a), photothermal characterization (b), and application of photothermal nonwovens (c). Production includes embedding melanin NPs onto pullulan nanofibers by coelectrospinning (PULL_M_ES) and coating (PULL_M_C). Photothermal characterization of PULL_M_ES was carried out under irradiation at 660 nm, and temperature was monitored with a thermal camera. The application in cultural heritage was assessed by placing the nonwoven wet by GVL on the surface to be cleaned under and without irradiation.

In this work, we propose to use an electrospun nonwoven embedded with photothermal melanin nanoparticles (NPs) to enhance the cleaning efficacy against highly insoluble materials, such as alkyd sprays. Furthermore, the proposed cleaning system is designed with environmental sustainability in mind. Melanin, obtained in this work from cuttlefish ink through a water centrifugation process, is a natural dark brown pigment composed of polymeric aromatic moieties aggregated together by hydrogen bonding, electrostatic, and π–π stacking interactions.^32−34^ Due to this structure, light-excited melanin preferentially undergoes nonradiative relaxation by releasing heat.^35,36^ Moreover, melanin-based nanosystems are of great interest because this material has been demonstrated to be very versatile for its biological functions,^37^ its roles in nanomedicine and material science,^38,39^ and its biocompatibility.^40^ In our system, the nonwoven is made of pullulan. This natural polysaccharide can be electrospun from an aqueous solution, eliminating the need for toxic organic solvents typically used in the electrospinning process.^41^ Lastly, in combination with the photothermal nonwoven, we employed γ-valerolactone (GVL), a well-known green solvent. Initially, melanin NPs were incorporated into the nonwoven using two methods: coelectrospinning with pullulan and coating preformed pullulan fibers (Figure 1a). Both types of materials were fully characterized, and the photothermal properties of the optimal system were investigated (Figure 1b). Through the use of red LED irradiation, the light-activated cleaning system was then tested on an alkyd varnish layer sprayed on a marble surface to simulate vandalism (Figure 1c). A very efficient removal of the varnish was observed within the irradiated area, demonstrating that the localized temperature increase combined with the solvent action excels in swelling and removing the cross-linked coating.

## Experimental Section

### Materials

γ-Valerolactone (GVL) was purchased from Sigma-Aldrich, and pullulan was purchased from TCI Europe. Melanin was obtained from cuttlefish ink using a centrifugation process. The alkyd spray “94, Twister Blue” paint was purchased from Montana Colors, and Ricci Marmi (Ravenna, Italy) kindly provided the marble.

### Extraction of Melanin NPs

Melanin NPs were obtained with a centrifugation method, similar to what is reported in the literature for melanin extraction from cuttlefish ink.^42,43^ Cuttlefish ink was extracted from the cuttlefish’s sack and diluted with Milli-Q water. The obtained ink dispersion was centrifuged at 3.000 rpm for 10 min to remove residues (sand or parts of the cuttlefish’s ink sack). After centrifugation, the supernatant was kept, diluted with Milli-Q water, and centrifuged at 6500 rpm for 10 min. Then, the supernatant was removed, and the pellets were washed three times with Milli-Q water by centrifugation. The obtained melanin dispersion was dried in the oven at 60 °C. Then, it was dispersed in the solvent, Milli-Q water or GVL, by stirring for 5 h to obtain a uniform 18 mg/mL dispersion.

### Fabrication of Nonwovens

A laboratory electrospinning machine (Spin-bow Lab Unit, Spinbow S.r.l., Italia) was employed to produce the nonwovens. The instrument is composed of a syringe containing the polymer solution connected to a steel needle with a blunt tip (internal diameter 0.51 mm) through a PTFE tube and an aluminum plate covered with a PTFE mask of (10 × 10 cm^2^ or 5 × 5 cm^2^), that was used as a collector. The distance between the needle and the collector was 18 cm, the voltage was 24–25 kV, and the flow rate of the polymer solution was set at 0.8 mL/h. Two types of nonwovens were fabricated by electrospinning: PULL sample was obtained starting from a solution of 19 w/v% pullulan in water; PULL_M_ES was obtained by electrospinning a solution of pullulan 19% w/V in a melanin NPs dispersion 18 mg/mL in water. In the latter case, the dispersion was sonicated for 10 min before electrospinning to prevent melanin NPs aggregation. A third nonwoven was prepared by soaking in a melanin NPs dispersion 18 mg/mL in GVL (PULL_M_C). The mat in the dispersion was first vortexed for 10 min and then left at rest for 20 min to ensure the most homogeneous coating. The nonwovens had a final thickness of 300–400 μm.

### Characterization Methods

Dynamic Light Scattering (DLS) was performed using the Malvern Nano Zetasizer instrument. Melanin NPs, sonicated for 10 min in water and GVL, have been placed in a plastic cuvette for the DLS analysis. Scanning electron microscope (SEM, Leica Cambridge Stereoscan 360) was performed at 20 kV accelerating on gold-sputtered samples. Melanin NPs 18 mg/mL dispersion in water and GVL have been sonicated for 10 min. Then, 2 μL of the dispersion was deposited on the SEM stub. The images have been processed with ImageJ,^44^ and the distribution of the nanoparticles’ diameters was determined by measuring about 200 NPs; the results are given as the average diameter ± standard error of the mean. The distribution of the nanofiber diameters was measured on almost 200 fibers and was obtained as average diameter ± standard deviation. Unidirectional ANOVA was used to test the statistical significance based on the difference between the average values (*p* < 0.001). Transmission Electron Microscopy TEM observations were performed using a Philips microscope with a voltage of 80 kV. A few fibers were electrospun directly on a TEM copper grid. Absorption spectra were obtained using the spectrophotometer PerkinElmer Lambda 650 in the wavelength range 300–900 nm. In order to quantify the amount of melanin in the sample PULL_M_ES, a calibration curve was obtained, measuring the absorption spectra of melanin dispersions in water at known concentrations, from 3.3 × 10^–3^ mg/mL to 3.5 × 10^–2^ mg/mL, and considering their absorbance at 850 nm. Three replicas of PULL_M_ES samples were dissolved in water (0.1 mg/mL), and absorption spectra were acquired to calculate the exact quantity of melanin in each sample. DSC measurements were performed with DSC Q2000 (TA Instruments), equipped with a Refrigerator Cooling system (RCS). Analyses were carried out under a 50 mL/min nitrogen flow: the thermal program consisted of a first heating ramp from −90 to 100 °C at a heating rate of 20 °C/min, a controlled cooling at 10 °C/min followed by a second heating scan. A TGA Q500 thermogravimetric analyzer (TA Instruments) was employed for thermogravimetric analysis by applying a heating ramp at 10 °C/min up to 800 °C under N_2_ atmosphere.

### Characterization of the Photothermal Behavior

Photothermal analyses were performed by irradiating the nonwoven with an LED at 660 nm (LZ1-10R2, Osram) (Figure S1). The LED, equipped with a lens (Thorlabs), irradiated an area of 5 × 5 mm^2^ and was placed 6 cm away from the sample. The nanofiber photothermal characterization was performed by irradiating a 1 × 1 cm^2^ tissue and monitoring the temperature increase in the irradiated area with a thermal camera (Optris Xi 400, IR camera, 80HZ frame rate, and 382 × 288 pixels of optical resolution) using the Optris PIX connect software. For each electrospun sample tested, the increase in temperature was recorded on the sample’s side, which was directly irradiated by the LED (front side) and subsequently on the sample’s back side. In particular, the tissue was tested at four LED irradiances, modulated by tuning the applied current: 223 W/m^2^ (0.1 A), 435 W/m^2^ (0.2 A), 648 W/m^2^ (0.3 A) and 858 W/m^2^ (0.4 A). Moreover, the performances were tested in 5 cycles for every irradiance, always on the front and back sides of the same tissue. Photothermal characterization of the nonwovens soaked with 2 μL of GVL was also carried out by irradiating at 648 W/m^2^. The cycles and the photothermal behavior in wet conditions measurements were replicated on three mats with the same area (1 × 1 cm^2^) and similar thickness (average thickness: 340 ± 10 μm).

### Graffiti Mock-Up Preparation

The mock-up was realized by spraying alkyd paint onto a marble slab. The mock-up was put in the stove at 70 °C for 48 h to ensure complete reticulation of the paint. Paint thickness was monitored by observing polished (500 up to 12000 grit size) mock-up cross sections embedded in resin and under an optical microscope using visible and UV light (Olympus Optical Microscope BX51, Tokyo, Japan).

### Cleaning Tests and Efficacy Evaluation

0.7 × 0.7 cm^2^ specimens were cut from the electrospun nonwoven for each cleaning test. The specimens were weighed, and their thickness was measured with a digital micrometre (Digimatic Micrometer, Mitutoyo Corporation). When GVL was used, the ratio between the volume of GVL and the mat’s weight was 2 μL/mg. This ratio was selected to have the solvent confined in the irradiation spot, increasing the cleaning spatial resolution. The sample was applied to the coated marble surface for 2 min. When irradiation was employed, the LED (irradiance 648 W/m^2^) was placed orthogonally to the sample at a distance of 6 cm, and the procedure was live-monitored with the thermocamera. After each test, three dry cotton swabs were rolled on the treated surface to remove paint residues. A digital microscope (AM4113T, Dino-lite) was employed for photographic documentation in visible light. The portable spectrophotometer CM-26dG/CM-26d (Konica Minolta) was used for the colorimetric analysis. Before each acquisition, the instrument was calibrated for stray light (Zero calibration) and reflectance (white calibration). The SCE (Specular Component Excluded) method was selected to acquire the measurements as it considers the sample’s morphology. The color and luminosity variations of the treated areas were evaluated regarding the colorimetric values of the marble (target). The colorimetric values measured for each treated area were averaged, and the Δ*E* (overall variation in the color space) and ΔL (luminosity variation) were calculated with respect to the average values of the marble. The calculations were made according to eqs 1–4, where *a*, *b* and *L* are the color coordinates of the target (t) and of the area treated (m).^45^
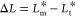
1
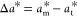
2
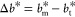
3
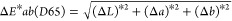
4

The hyperspectral camera Specim IQ (VNIR 400–1000 nm (CMOS)) was utilized to capture a hyperspectral image, where each pixel represented a unique spectrum 400–1000 nm with a spatial resolution of 300 μm positioning the camera at a distance of 15 cm from the sample. The data were elaborated in MatLab by mapping the ratio of the most intense spray paint peak (610 nm) and the baseline (920 nm). Spectra from the marble, spray paint, and cleaned area were extracted from single pixels.

## Results and Discussion

### Production and Characterization of Electrospun Nonwovens

Melanin NPs, extracted from cuttlefish ink, were dispersed in either water or GVL to prepare photothermal pullulan-based electrospun materials. Both dispersions displayed good stability for almost 1 h; then, a few precipitates appeared at the bottom. The dispersions were sonicated every hour during their use to limit the formation of aggregates. The two dispersions were characterized by SEM and DLS (Figure S2 and Table 1). SEM shows NPs with a diameter of almost 150 nm in both the dispersions (151 ± 17 nm and 155 ± 17 nm in water and GVL, respectively). However, in GVL, NPs appear more aggregated than in water. This result aligns with DLS analysis, in which NPs in water and GVL have hydrodynamic diameters of 205 nm (PDI = 0.08) and 281 nm (PDI = 0.5), respectively. The increased aggregation in GVL is attributed to the negatively charged surface of melanin NPs (ζ-potential of cuttlefish melanin NPs is approximately −30 mV^46^), which reduces their stability in less polar solvents.

**Table 1:** Cleaning Tests Performed with the Indication of the Entry Number, the Type of Nonwoven, and the Use of Solvent and Irradiation

Entry	type of nonwoven	use of GVL	use of irradiation
1	PULL	yes	no
2	PULL	no	yes
3	PULL	yes	yes
4	PULL_M_ES	yes	no
5	PULL_M_ES	no	yes
6	PULL_M_ES	yes	yes

Two approaches were employed to impart photothermal properties to the pullulan ES mats. In one approach, pullulan was dissolved in the water NPs dispersion and directly electrospun to gain fibers embedding melanin NPs (sample code: PULL_M_ES). In the second case, pullulan was electrospun from pure water (sample code: PULL), and the fibers were subsequently coated with melanin NPs by soaking the nonwoven in the NPs dispersion in GVL (sample code: PULL_M_C) (Figure 1a).

**Figure 2:**
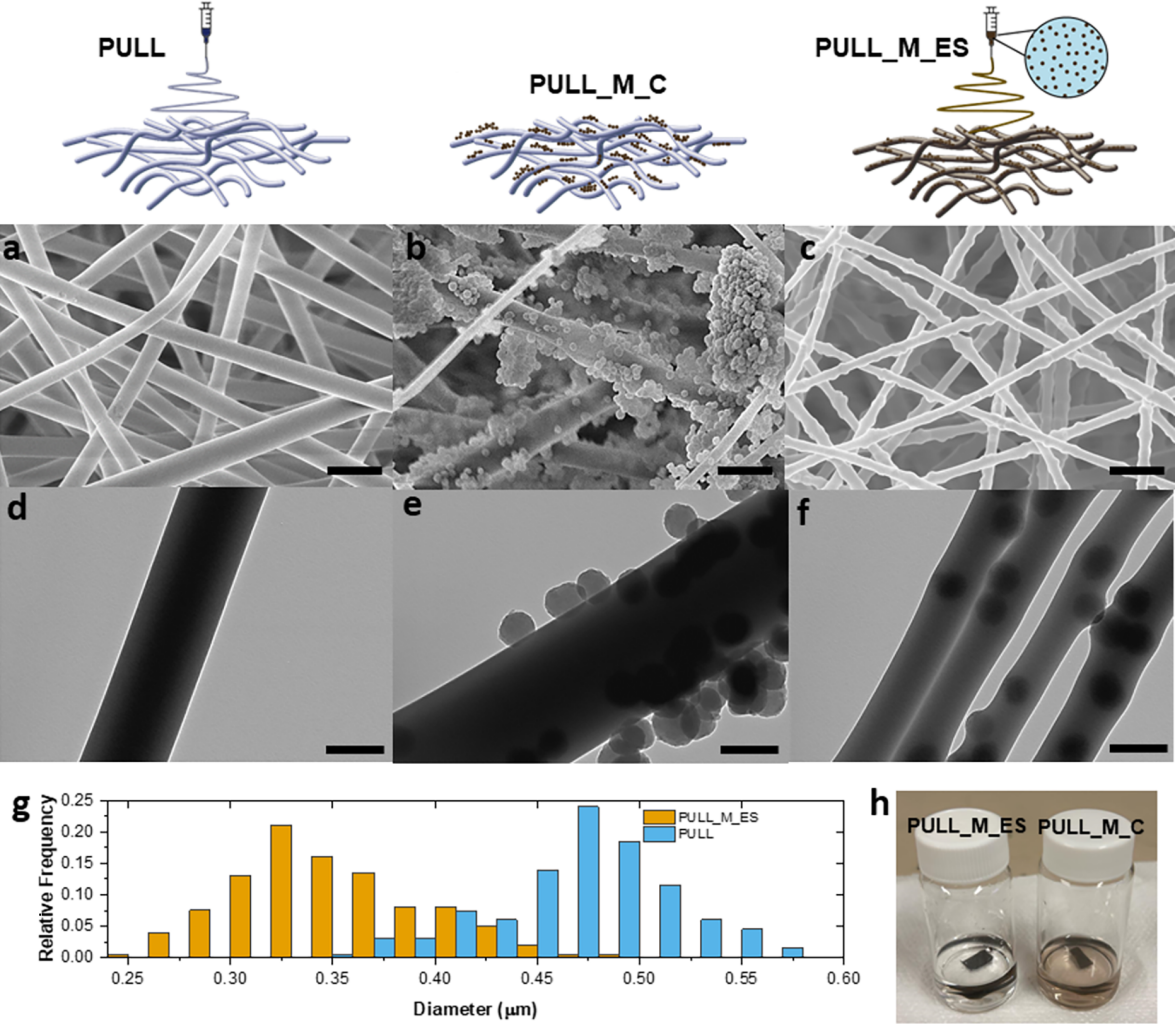
SEM micrographs (scale bars = 1 μm) of PULL (a), PULL_M_C (b), and PULL_M_ES (c). Additional SEM images are reported in Figure S3. TEM micrographs (scale bars = 200 nm) of PULL (d), PULL_M_C (e), and PULL_M_ES (f). Additional TEM images are reported in Figure S4. (g) Fiber diameter distribution of PULL (light blue) and PULL_M_ES (yellow). (h) Melanin NPs release in GVL from PULL_M_ES and PULL_M_C: images are taken 10 min after the immersion of the samples in GVL.

SEM micrographs (Figure 2a) show that PULL is characterized by bead-free fibers with a continuous structure and regular diameters (0.47 ± 0.04 μm, Figure 2g). In the PULL_M_C sample, nanoparticles coated on the fiber surface are clearly visible and tend to form agglomerates (Figure 2b and Figure S3a–c). In contrast, the morphology of the PULL_M_ES sample is characterized by frequent beads along the fibers, attributed to the encapsulated melanin nanoparticles within the fibers themselves (Figure 2c and Figure S3d–f). The average fiber diameter is 0.35 ± 0.04 μm (Figure 2g). The difference in diameter between PULL and PULL_M_ES could be ascribed to different electrical conductivity of the two solutions used for electrospinning, given the presence of the melanin NPs, which are negatively charged at neutral pH.^47^ Indeed, in the electrospinning technique, solutions with higher electrical conductivity lead to a decrease in fiber diameter.^48^ TEM analysis (Figure 2 and Figure S4) further elucidates fiber structure and NPs distribution: PULL fibers (Figure 2d) are continuous and homogeneous, PULL_M_C fibers (Figure 2e and Figure S4a–d) are rich in melanin NPs at their surface, in PULL_M_ES (Figure 2f and Figure S4e–h) melanin NPs appear to have been incorporated within the nanofibers of the nonwoven fabric.

Based on the morphological characterization, it is reasonable to expect that PULL_M_C will raise the temperature of GVL, chosen as the green solvent for the varnish cleaning procedure, more effectively upon irradiation compared to PULL_M_ES. Indeed, in PULL_M_C, the photothermal NPs would be directly in contact with GVL, whereas, in PULL_M_ES, the NPs are embedded in an insulating, albeit thin, polymer matrix. However, the first aspect to consider is the possible leaching of melanin NPs from the nonwoven in the presence of GVL. It is crucial to prevent the release of NPs, as they could remain as impurities on the cleaned surface, changing its aesthetical appearance. The stability of NPs in PULL_M_ES and PULL_M_C was evaluated by submerging the samples in GVL. After 10 min of soaking, GVL in contact with PULL_M_C turned from colorless and transparent to opaque and brownish, whereas the GVL in contact with PULL_M_ES maintained its clarity and lack of coloration (Figure 2h). This qualitative test demonstrated that the direct spinning of the polymer in the NPs dispersion allows the production of a more stable and homogeneous fabric. At the same time, the surface interactions between melanin NPs and pullulan fibers are not strong enough to prevent NPs from leaching if the melanin dispersion is applied as a coating on the pullulan nonwoven. For this reason, PULL_M_C was not further characterized and tested.

The quantification of melanin NPs in the PULL_M_ES mats was determined by dissolving the PULL_M_ES mat in water, measuring the absorption spectra of the so-obtained melanin NP dispersions, and calculating NP concentration based on a calibration curve (slope: 3.82 A_850 nm_ mL/g, *R*^2^: 0.999, Figure S5). The average melanin concentration obtained with this method is 7.8 ± 0.2 wt %, not far from the expected value of 8.7 wt %, calculated considering the amount of NPs employed during the spinning process.

### Photothermal Behavior

**Figure 3:**
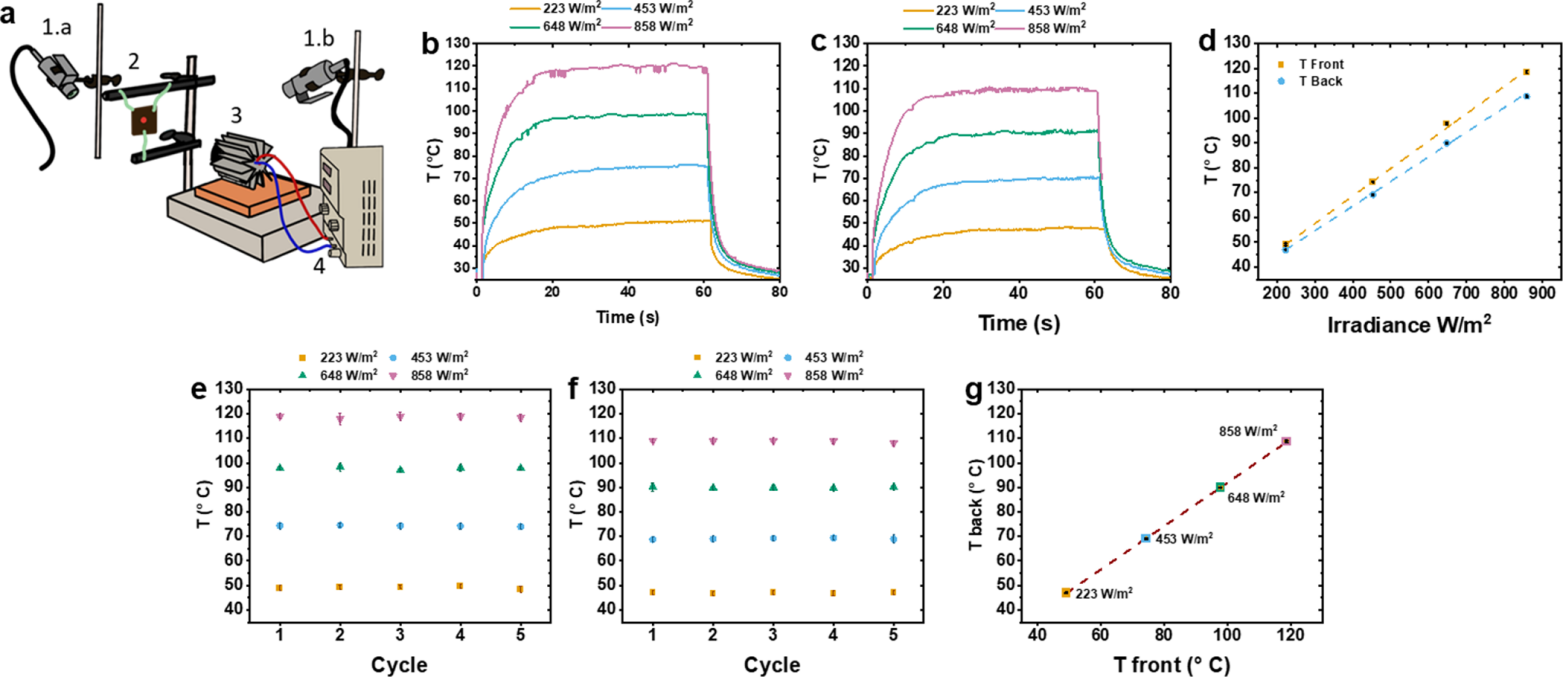
(a) Scheme of the setup employed for the monitoring of the photothermal properties of PULL_M_ES composed of: (1) thermal camera (1.a: back side; 1.b: front side monitoring); (2) PULL_M_ES sample; (3) LED; (4) power supply. Temperature variation over time during irradiation of PULL_M_ES at different irradiances: (b) temperature of the front side and (c) of the back side (the arrow indicates the moment when the LED is switched on and switched off). (d) Linear fitting of the maximum temperatures recorded after 1 min of irradiation as a function of LED irradiance monitored on the backside (blue) and front-side (yellow) of the PULL_M_ES sample. The standard deviation is in the range of 0.2–0.5 °C. Maximum temperatures of PULL_M_ES reached along five subsequent irradiation cycles for each LED irradiance: (e) front side monitoring and (f) back side monitoring. (g) Comparison between the maximum temperatures (obtained by the average of the average plateau temperature of each cycle) reached for every irradiance at the front (T Front) and the back (T back) (standard deviations are reported in Table S2).

The photothermal properties of PULL_M_ES were evaluated through the setup depicted in Figure 3a. The nonwoven was irradiated at 660 nm, with a red LED selected for its high efficiency in promoting the nonradiative decay of melanin in this wavelength range,^49^ as well as for being a cost-effective and safe light source. The temperature change at four irradiances (223 W/m^2^, 453 W/m^2^, 648 W/m^2^, 858 W/m^2^) was monitored by a thermal camera, either on the irradiated side (“front side”, camera in position 1.b) or on the opposite side (“backside”, camera in position 1.a).^48^ Once irradiated, PULL_M_ES reaches its maximum temperature in almost 30 s, both on the front and back sides, and remains stable until the light is on, while a rapid temperature decrease is observed as soon as the light is switched off (Figure 3b,c). In Figure 3d, the average plateau temperature was plotted for each irradiance, showing that the temperature increases as the irradiance increases, with a linear correlation for both the front (slope: 0.13 °C m^2^/W, *R*^2^: 0.97) and the back (slope: 0.07 °C m^2^/W, *R*^2^: 0.99). However, as previously observed in a similar system,^50^ the maximum temperatures monitored on the backside are lower than the front when the mat is irradiated with the same irradiance. This result can be ascribed to the presence of air in the mat pores, which favors the scattering of radiation to the surface of the fibers at the expense of absorption. Furthermore, the low thermal conductivity of air contributes to thermally insulating the furthest side from irradiation.^51^

The photothermal response of PULL_M_ES was also evaluated under five cycles of light irradiation for every irradiance previously tested. Figure 3e,f show that both the front and the back sides of PULL_M_ES nonwoven maintain the same photothermal response, highlighting that the material does not undergo any degradation or modification of its photothermal behavior, even after five cycles of light irradiation at different irradiances (Table S2). As previously discussed, the maximum temperatures observed at the front are higher than the ones observed at the back. Interestingly, there is a linear correlation between the front and back sides (Figure 3g, slope: 0.68, *R*^2^: 0.97).

**Figure 4:**
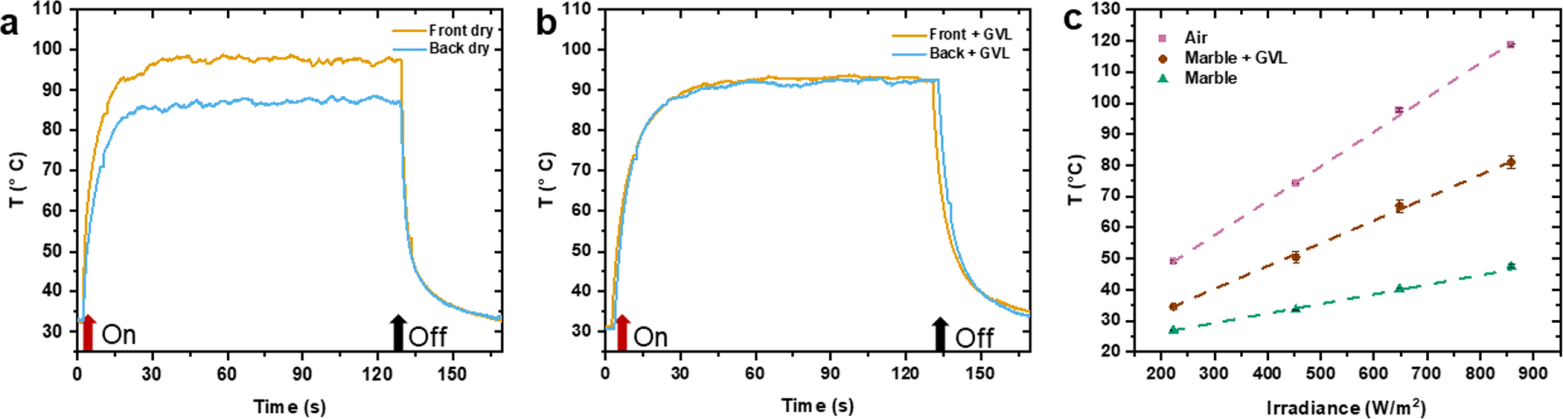
Temperature increase during irradiation on the front side and back side of PULL_M_ES: (a) dry nonwoven and (b) nonwoven wet by GVL (irradiance = 640 W/m^2^, the arrow indicates the moment when the LED is switched on and switched off). (c) Temperature monitoring at different irradiances of PULL_M_ES (front side), in the air (pink), deposited on marble (green), and wetted by GVL and deposited on marble (brown).

Since the temperature reached by the mat depends not only on the irradiation conditions but also on the medium in contact with it, the photothermal behavior of PULL_M_ES was also investigated in the presence of GVL (Figure 4). When the dry PULL_M_ES is irradiated, as already observed, the temperature rises differently on the front and the back sides (Figure 4a). Conversely, in the presence of GVL, the temperature on both the front and back sides becomes uniform and falls between the temperatures observed on the front and back sides of the dry sample (Figure 4b). This effect is likely due to the higher thermal conductivity of the solvent (organic solvents have thermal conductivities in the range 0.1–0.17 W/mK)^52^ compared to air (0.025 W/mK),^51^ resulting in more uniform heat distribution throughout the nonwoven.

Intending to apply the PULL_M_ES for graffiti removal, we measured the temperature reached by the nonwoven when in contact with marble. More specifically, we compared the temperature increase at the front side as a function of the irradiance (Figure 4c) under different conditions: dry sample in air, dry sample in contact with marble on the back side, and sample wetted by GVL and in contact with marble on the back side, the latter representing the conditions of the cleaning application. Dry PULL_M_ES in contact with marble exhibited a lower maximum temperature and a slower temperature increase with irradiance than the dry sample in the air. This outcome is expected because marble has a higher thermal conductivity (2.8–3 W/mK)^53^ than air. The nonwoven wetted by GVL and in contact with marble displayed an intermediate behavior compared to the other two cases. Under these conditions, the thermal conductivity of the solvent, which is higher than that of the air but lower than that of the marble, contributes to a more uniform heat distribution.

### Cleaning Tests

The cleaning tests were performed on a mock-up where the alkyd spray paint was spread on a marble surface to gain a 25 ± 2 μm thick layer (Figure S6). Preliminary tests were conducted to set the cleaning conditions using PULL_M_ES wetted with GVL. Due to the high thermal conductivity of marble, we expected that the temperature reached on its surface would be lower than what is observed on the forefront of the irradiated mats. Since marble is a stable material, it is out of the purpose of this paper to understand the exact temperature reached on its surface, while the aim is to define the irradiance conditions to remove the alkyd layer applied on it efficiently. To this aim, different irradiance conditions (223 W/m^2^, 453 W/m^2^, 648 W/m^2^) were tested by putting the wetted mats in contact with the alkyd spray applied on the marble mock-ups for 2 min, which, after preliminary tests at different times of application, was defined as a reasonable time to accomplish the cleaning treatment and to achieve reproducible results. The tests carried out in triplicate highlighted that the complete removal of the alkyd layer was achieved with irradiation at 648 W/m^2^ for 2 min, corresponding to a temperature increase in the forefront of about 65 °C.

**Figure 5:**
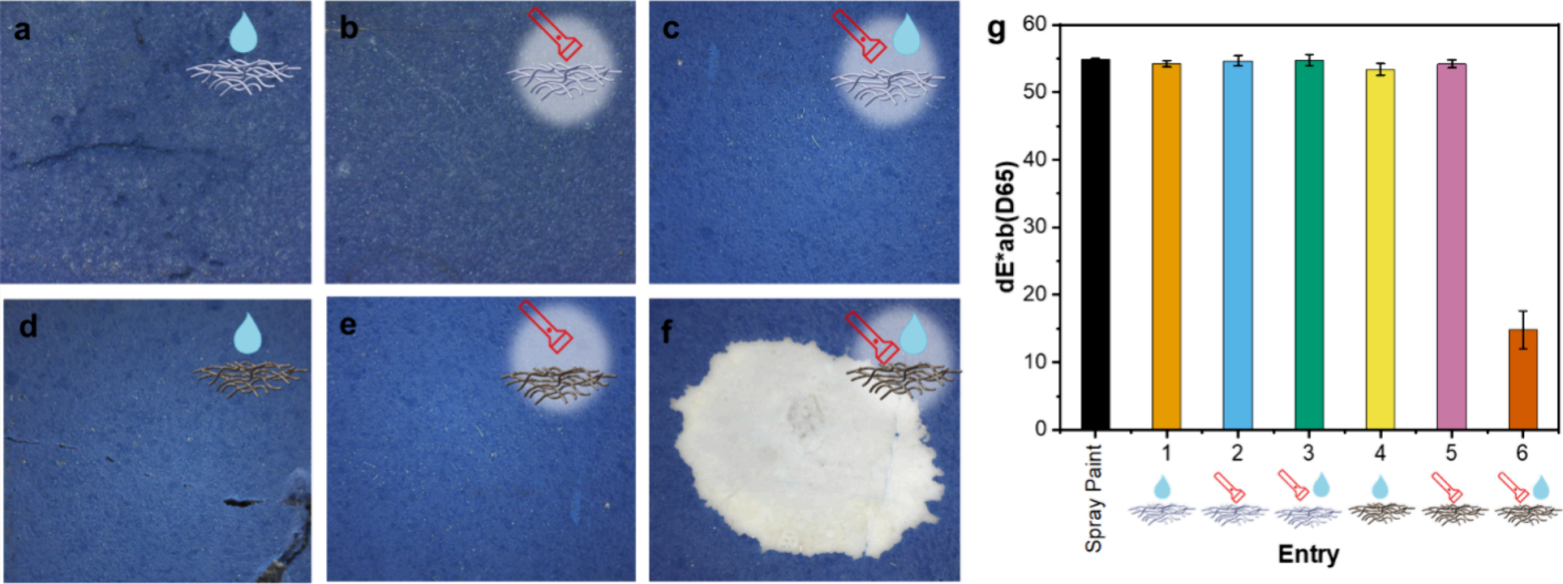
(a–f) Photographs acquired with DinoLite digital microscope in visible light. Tests performed with (a) PULL without irradiation and with GVL solvent (entry 1), (b) PULL without GVL and with irradiation (entry 2), (c) PULL with GVL and with irradiation (entry 3), (d) PULL_M_ES without irradiation and with GVL solvent (entry 4), (e) PULL_M_ES with irradiation and without GVL solvent (entry 5), and (f) PULL_M_ES with irradiation and with GVL solvent (entry 6). (g) Results of the colorimetric measurements performed on the treated areas: variation in the color space.

These latter conditions were thus employed to carry out quantitative tests to highlight the role of the photothermal effect on the cleaning efficacy. More specifically, six experiments were performed, as listed in Table 1, that differ in the type of nonwoven (either PULL or PULL_M_ES), the use of GVL and the use of irradiation (Figure 5).

As shown in Figure 5a–g, the alkydic layer is removed only when PULL_M_ES is irradiated in the presence of GVL, reaching a front side temperature of 65 °C (entry 6, Figure 5f). Colorimetric measurements aim to evaluate chromatic variations by using the values of the underlying marble as a reference (Figure 5g). Therefore, the smaller the difference, the closer the treated area’s values in the color space are to the original strata to be preserved. In the only test where a visibly effective cleaning was achieved (entry 6), the dE*ab reached a level near 10 (a value considered the threshold for two colors to be distinguishable by the human eye).^45^

**Figure 6:**
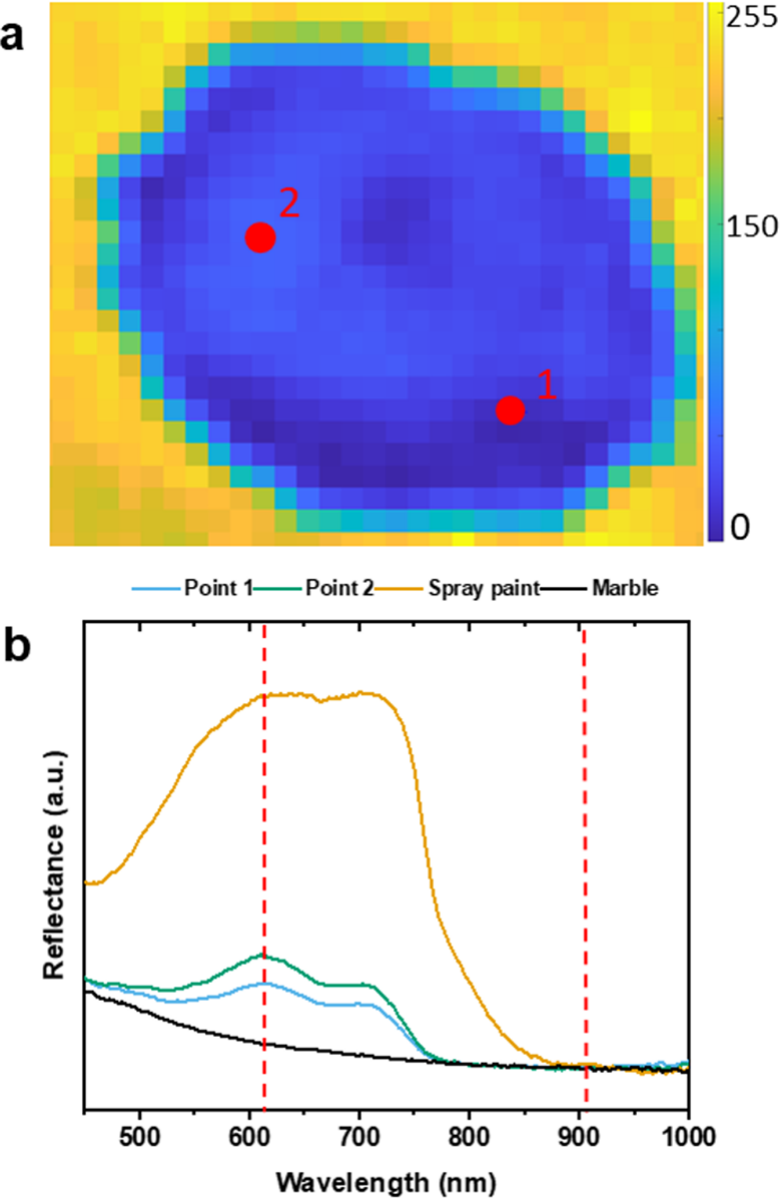
(a) Map of the ratio *I*_610 nm_/*I*_920 nm_ of the spectra acquired with the HSI camera with the indication of the color scale and the two pixels extracted from the cleaned area. (b) Spectra extracted from the pixels of marble (black), Spray paint (yellow), point 1 (light blue), and point 2 (green) with the indication of the two peaks employed for the ratio (red dashed lines).

Hyperspectral imaging was employed to evaluate further the cleaning performance. Figure 6a shows the results obtained by mapping the ratio of the most intense spray paint peak (610 nm) to the baseline (920 nm). This approach was chosen to reduce errors associated with baseline differences. By extracting two spectra from the map, a weak signal can be observed from the residue of the blue pigment, probably partially absorbed into the porous marble surface (Figure 6b). Nonetheless, considering the visual appearance, the colorimetric and the hyperspectral imaging results, the cleaning efficacy is considered more than acceptable.

Considering all the cleaning conditions tested, it can be concluded that the combined action of GVL and the increased temperature allows the reticulated alkyd spray paint to swell rapidly, enabling its removal with dry cotton swabs. At the employed irradiance conditions, we expect that the temperature reached on the alkyd layer is above the polymer *T*_g_ (about 24 °C, Figure S7), thus leading to an increase of the chain mobility and, consequently, free volume, which facilitates solvent diffusion within the polymer chains, further increasing the free volume. These combined phenomena enhance film plasticity, making removing the coating easier and quicker.^54−57^

## Conclusions

In this work, we report the development of a photothermal nonwoven based on incorporating melanin NPs in pullulan electrospun nanofibers. Their fabrication is performed in one step by embedding the NPs in the fibers during electrospinning. Compared to melanin NPs coating on the nonwoven, this method guarantees a uniform distribution and excellent stability of the NPs inside the fibers. Moreover, using cuttlefish ink-derived melanin NPs and water as the electrospinning solvent allows for an entirely sustainable process. This composite nonwoven is a light-responsive system in which a few seconds of red-light irradiation generates an outstanding photothermal effect, going from room temperature to more than 100 °C in 30 s, with exceptional resistance to multiple irradiation cycles. These photothermal nonwovens, combined with a green solvent (GVL), are employed as a sustainable and nontoxic technique to remove alkyd insoluble varnishes used in vandalism. The effectiveness of the removal process relies on the combined action of the GVL solvent and the photothermal effect. The solvent causes swelling of the varnish, while the photothermal effect further increases the mobility of the alkyd polymer chains, enabling their removal in a rapid process with minimal solvent use. Furthermore, the solvent and photothermal effect combination allows for a cleaning confined only within the irradiated area. The possibility of a precise removal with high spatial resolution is an added value that also permits other applications of this technique in which this kind of control is required. Based on these observations, the proposed materials offer a promising approach to street art conservation. A significant challenge in this domain lies in the selective removal of vandalism from street art, as both the artwork and the unwanted additions often utilize similar materials. While the proposed materials demonstrate potential, further optimization is necessary to minimize the maximum temperature reached during the treatment. This optimization will ensure the preservation of the underlying paint layers of the artwork.

## Supporting Information Available

LED emission properties, characterization of melanin NPs dispersions in water and GVL (DLS analysis and SEM images), further morphological characterization of electrospun nonwovens incorporated with melanin (SEM and TEM images), melanin quantification in electrospun nonwovens (absorption spectroscopy and DLS analysis), further characterization of photothermal properties of melanin electrospun nonwovens, optical microscope micrograph of the mock-up cross-section, and DSC of the alkyd resin (PDF)
