# Supplementary material for: Nanofibrous Photothermal Materials from Natural Resources: A Green Approach for Artwork Restoration
Source: ACS Appl Mater Interfaces. 2024 Dec 6;16(50):69829–38. doi: 10.1021/acsami.4c14532 (PMC11660143; doi:10.1021/acsami.4c14532)
Supplement: Supplementary file 1 — am4c14532_si_001.pdf [file am4c14532_si_001.pdf]

Supporting information

Nanofibrous photothermal materials from natural resources: a green approach for artwork restoration

Arianna Menichetti[1,2,3,‡], Francesca Ramacciotti[1,‡], Giorgia Sciutto[1], Maria Letizia Focarete[1,2,4], Marco Montalti[1,2,3,4], Silvia Prati[1]*, Chiara Gualandi[1,2,5]*

[1]Department of Chemistry "Giacomo Ciamician", University of Bologna, Via Selmi 2, 40126 Bologna, Italy

[2]INSTM UdR of Bologna, University of Bologna, Via Selmi 2, 40126 Bologna, Italy

[3]Department of Chemistry "Giacomo Ciamician", University of Bologna, Via Dario Campana 71, 47922 Rimini, Italy

[4]Health Sciences & Technologies (HST) CIRI, University of Bologna, Via Tolara di Sopra 41/E, 40064 Ozzano Emilia Bologna, Italy

[5]Interdepartmental Center for Industrial Research on Advanced Applications in Mechanical Engineering and Materials Technology, CIRI-MAM, University of Bologna, Viale Risorgimento, 2, 40136 Bologna, Italy

Corresponding Authors: Prof. Silvia Prati, e-mail: s.prati@unibo.it. Prof. Chiara Gualandi, e-mail: c.gualandi@unibo.it.

‡ A.M. and F.R. contributed equally

1. *Emission light of the LED source* 2
2. *Characterization of melanin NPs* 2
3. *Morphological characterization of electrospun non-wovens* 3
4. *Melanin quantification in the pullulan nanofibres (PULL_M_ES)* 4
5. *Photothermal characterization of PULL_M_ES* 4
6. *Cleaning tests* 5
7. *References* 5

1. Emission light of the LED source

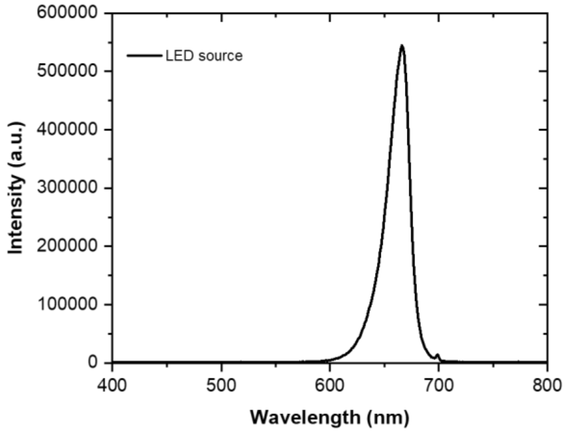

Figure S1. Emission spectrum of the LED source (660 nm) used for irradiation.

2. Characterization of melanin NPs

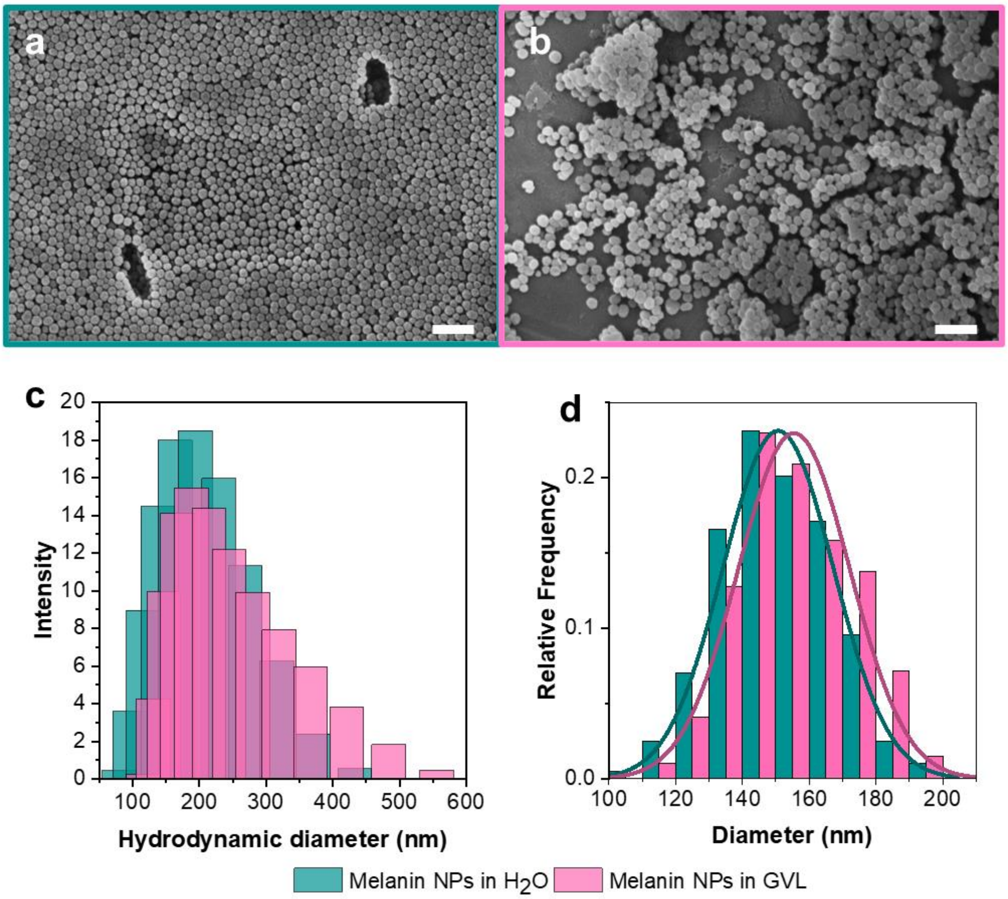

Figure S2. SEM micrographs (scale bar = 600 nm) of melanin NPs obtained from dispersions in water (a) and GVL (b). (c) Hydrodynamic diameter distribution of melanin NPs dispersions in water (pink) and GVL (green), obtained by DLS analysis. (d) Diameter distribution of melanin NPs in water (pink) and GVL (green) obtained by SEM images.

Table S1. Diameter distribution of melanin NPs in water and GVL obtained by SEM and DLS.

Melanin NPs dispersion	Diameter by SEM [nm]	Hydrodynamic diameter by DLS [nm]
Water	151 ± 17	205 (PDI 0.08)
GVL	155 ± 17	281 (PDI 0.5)

3. Morphological characterization of electrospun non-wovens

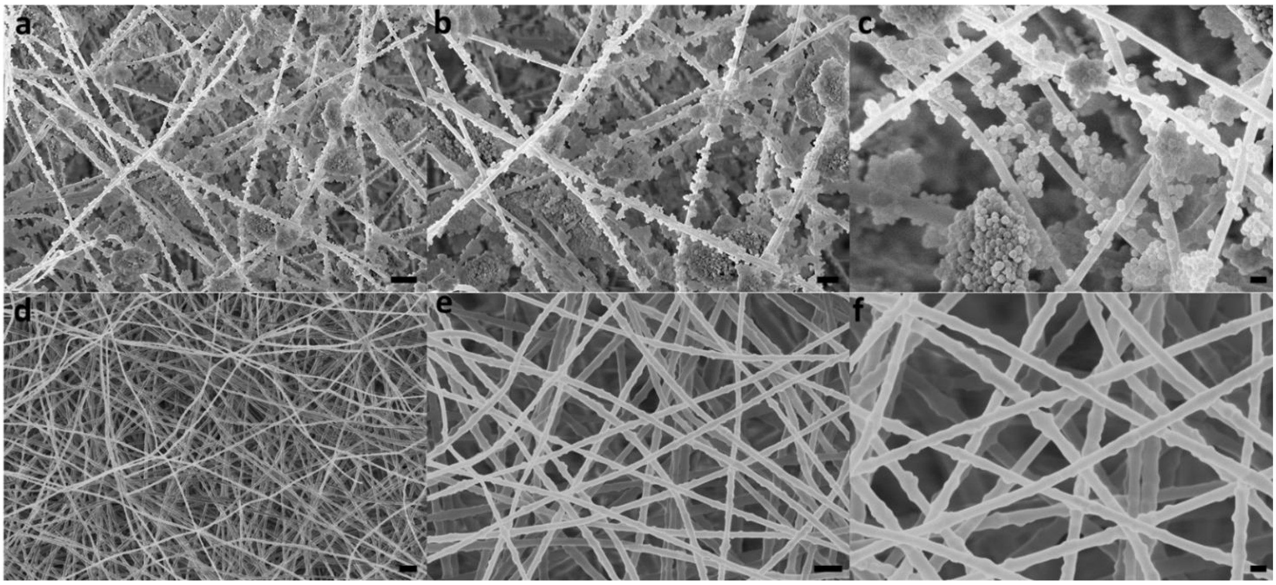

Figure S3. SEM images of PULL_M_C (a-c) and PULL_M_ES (d-f). Scale bar: 2 µm (a and d); 1 µm (b and e); 400 nm (c and f).

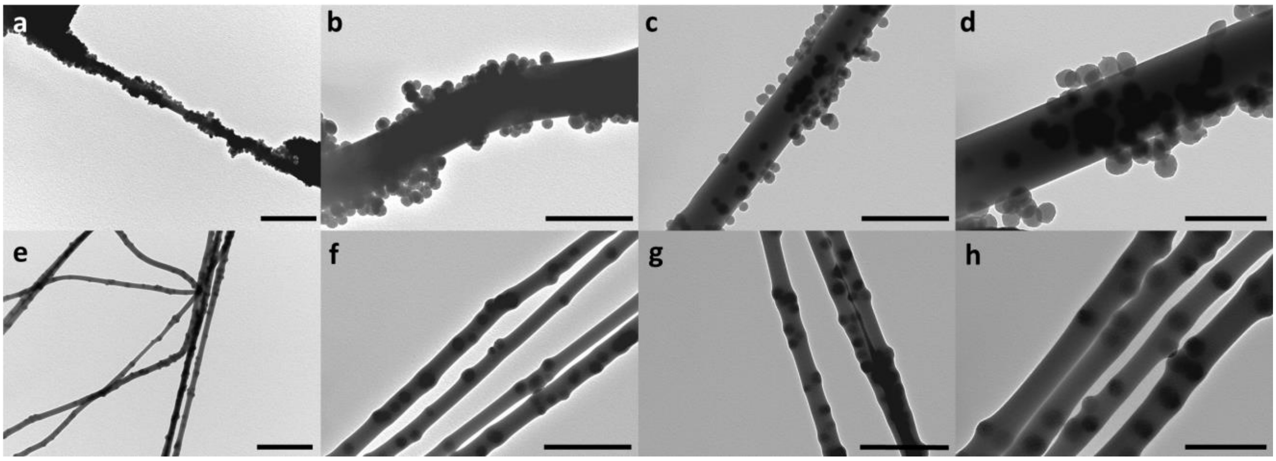

Figure S4. TEM images of PULL_M_C (a-d) and PULL_M_ES (e-h). Scale bar: 2 µm (a and e); 1 µm (b, c, f and g); 500 nm (d and h).

4. Melanin quantification in the pullulan nanofibres (PULL_M_ES)

A calibration curve (Figure S5a) was obtained by measuring the absorption spectra of melanin dispersions in water at known concentrations and considering their absorbance at 850 nm (Figure S5b). This wavelength was chosen to minimize the different scattering contributions to the absorption spectra of the melanin dispersions used for the calibration curve and the melanin dispersions obtained after the solubilization of PULL_M_ES. Indeed, DLS analysis revealed that the melanin NPs dispersed in water have a mean diameter of 200 nm, whereas, in the dispersion obtained after the solubilization of PULL_M_ES, particles have a larger mean diameter (290 nm) (Figure S5c), probably due to a higher aggregation they have in the mat. This difference in size, being Rayleigh scattering directly correlated to the sixth power of the particle's radius,[1] leads to different scattering signals in the absorption spectra. Due to the inverse proportionality of Rayleigh scattering and wavelength,[1] at large wavelengths, as 850 nm, the scattering contribution is neglectable with respect to the absorbance of melanin, making possible its quantification based on the calibration curve.

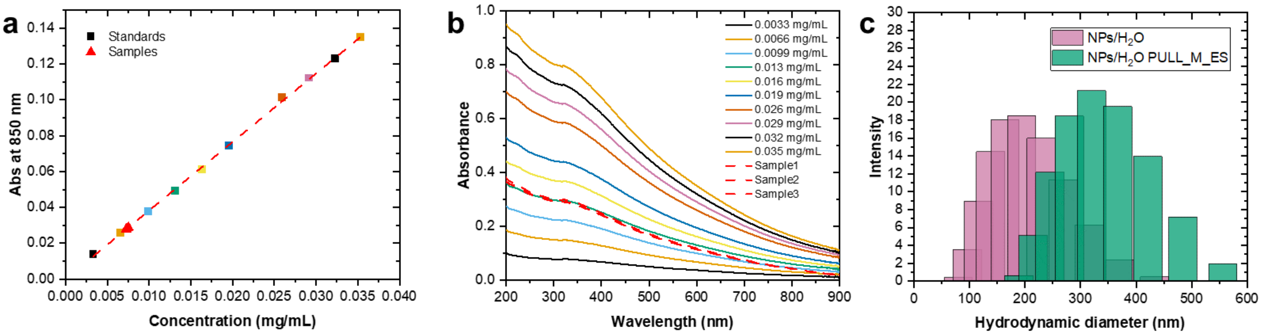

Figure S5: Calibration curve (a) and absorption spectra (b) for calculating the amount of melanin NPs embedded in PULL_M_ES. (c) DLS analysis of melanin NPs water dispersion and melanin NPs water dispersion after PULL_M_ES dissolution.

5. Photothermal characterization of PULL_M_ES

Table S2. PULL_M_ES was irradiated by the LED source at four different irradiances, and the temperature was monitored over time on both the front and back sides of the sample. Average plateau temperature reached at different irradiances on five cycles of irradiation.

Irradiance [W/m^2]	Front side temperature [°C]	Back side temperature [°C]
223	49.1 ± 0.5	46.9 ± 0.2
453	74.3 ± 0.2	69.1 ± 0.3
648	97.8 ± 0.5	89.9 ± 0.2
858	118.6 ± 0.5	108.8 ± 0.4

6. Cleaning tests

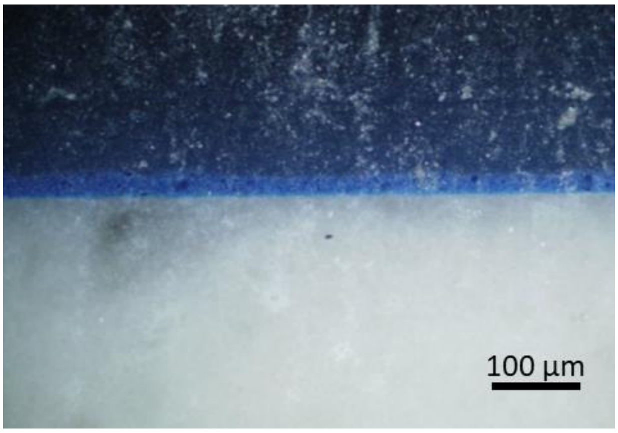

Figure S6. Optical microscope micrograph (20x) of the mock-up cross-section embedded in resin.

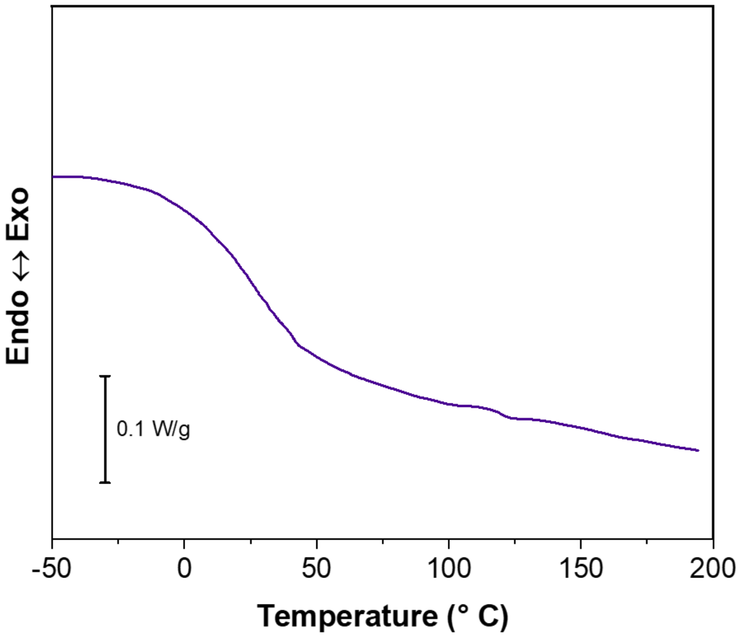

Figure S7. DSC first heating scan of the alkyd resin after curing for 48 h at 70 °C.

7. References

(1) Potter, K. S.; Simmons, J. H. Chapter 3 - Optical properties of insulators-fundamentals. In *Optical Materials (Second Edition)*, Potter, K. S., Simmons, J. H. Eds.; Elsevier, **2021**; pp 101-171.
